# Supplementary material for: Robust and bias-free localization of individual fixed dipole emitters achieving the Cramér Rao bound for applications in cryo-single molecule localization microscopy
Source: PLoS One. 2022 Feb 4;17(2):e0263500. doi: 10.1371/journal.pone.0263500 (PMC8815875; doi:10.1371/journal.pone.0263500)
Supplement: S1 File — The supplementary information contains S1 Table showing the simulation parameters, and S1–S10 Figs. (PDF) [file pone.0263500.s001.pdf]

Supplementary Information

Robust and bias-free localization of individual fixed dipole emitters achieving the Cramér Rao bound for applications in cryo-single molecule localization microscopy

Fabian Hinterer[1+], Magdalena C. Schneider[2+], Simon Hubmer[3], Montserrat López-Martinez[2], Philipp Zelger[4], Alexander Jesacher[4], Ronny Ramlau[1*], and Gerhard J. Schütz[2*]

[1]Institute of Industrial Mathematics, Johannes Kepler University Linz, Linz, Austria
[2]Institute of Applied Physics, TU Wien, Vienna, Austria
[3]Johann Radon Institute Linz, Linz, Austria
[4]Division for Biomedical Physics, Medical University of Innsbruck, Innsbruck, Austria
[*]ronny.ramlau@jku.at (RR), schuetz@iap.tuwien.ac.at (GJS)
[+]these authors contributed equally to this work

Fig.	ϕ	Astigm.	N	Reduced excitation	b	Angle noise θ	Angle noise ϕ	Pixel size	ROI
1	$0, \pi/4$	no	$5 \cdot 10^5$	no	0	—	—	108 nm	25×25
2	$\pi/4$	no	$5 \cdot 10^5$	no	300	0°	0°	108 nm	17×17
3	$\pi/4$	yes	$5 \cdot 10^5$	no	100	0°	0°	108 nm	17×17
4	$\pi/4$	yes	$5 \cdot 10^5$	no	0	0°	0°	108 nm	17×17
5	$\pi/4$	yes	$5 \cdot 10^5$	no	100	0°	0°	108 nm	17×17
6	$\pi/4$	yes	$5 \cdot 10^5$	no	0, 300	0°	0°	108 nm	various
7	$\pi/4$	yes	$5 \cdot 10^5$	no	100	2°	2°	108 nm	17×17
8	$\pi/4$	yes	$5 \cdot 10^5$	yes	100	2°	2°	108 nm	17×17
S1	$\pi/4$	yes	$5 \cdot 10^5$	no	0	0°	0°	108 nm	17×17
S2	$\pi/4$	no	$5 \cdot 10^5$	no	100	0°	0°	108 nm	17×17
S3	0	yes	$5 \cdot 10^5$	no	0	0°	0°	108 nm	17×17
S4	$\pi/4$	yes	$5 \cdot 10^4$, $5 \cdot 10^3$	no	0	0°	0°	108 nm	17×17
S5	$\pi/4$	yes	$5 \cdot 10^5$	no	0	0°	0°	216 nm	17×17
S6	$\pi/4$	yes	$5 \cdot 10^5$	no	300	0°	0°	108 nm	17×17
S7	$\pi/4$	yes	$5 \cdot 10^5$	no	300	2°	2°	108 nm	17×17
S8	$\pi/4$	yes	$5 \cdot 10^5$	no	100	4°	2°	108 nm	17×17
S9	$\pi/4$	yes	$5 \cdot 10^5$	no	300	4°	2°	108 nm	17×17
S10	$\pi/4$	yes	$5 \cdot 10^5$	yes	0	0°	0°	108 nm	17×17

Table S1

Table of simulation parameters. The inclination angle was set to $\theta = \pi/2, \pi/3, \pi/6, 0$. If not mentioned otherwise, 1000 simulations per data point were performed. Columns: Figure number, ϕ azimuthal angle, astigmatism, N number of photons, reduced excitation, b background noise, standard deviation of estimation of θ, standard deviation of estimation of ϕ, pixel size, region of interest for fit.

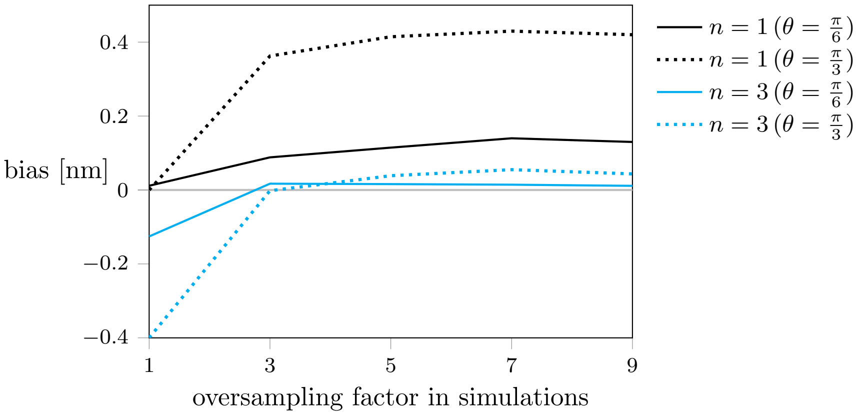

Figure S1

Discretization of the PSF. Localization bias arising from the choice of the oversampling factor in the calculation of the PSF. An oversampling factor of 1 corresponds to the PSF being evaluated at the discrete positions of the camera pixels. For larger oversampling factors, the PSF was evaluated on a finer grid, and resulting subpixel values were subsequently summed up. The x-axis shows the oversampling factor used for the simulation of the PSF. For the fitting, we used a PSF model calculated with an oversampling factor of n (black lines indicate $n = 1$, and blue lines $n = 3$). The defocus was set to $d = -500$ nm. Each data point represents 1500 simulations. A list of the remaining simulation parameters is given in Table S1.

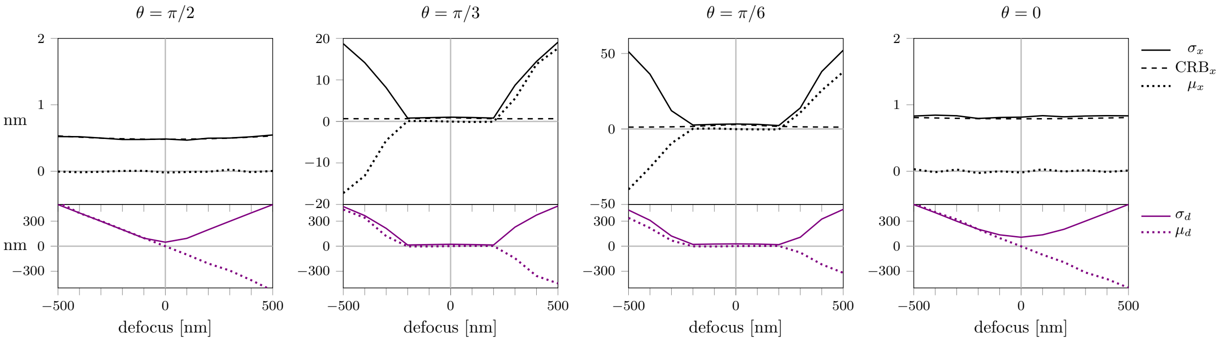

Figure S2

Localization errors without astigmatism. All parameters were identical to Fig. 2, except background noise, which was reduced to $b = 100$. A list of all simulation parameters is given in Table S1.

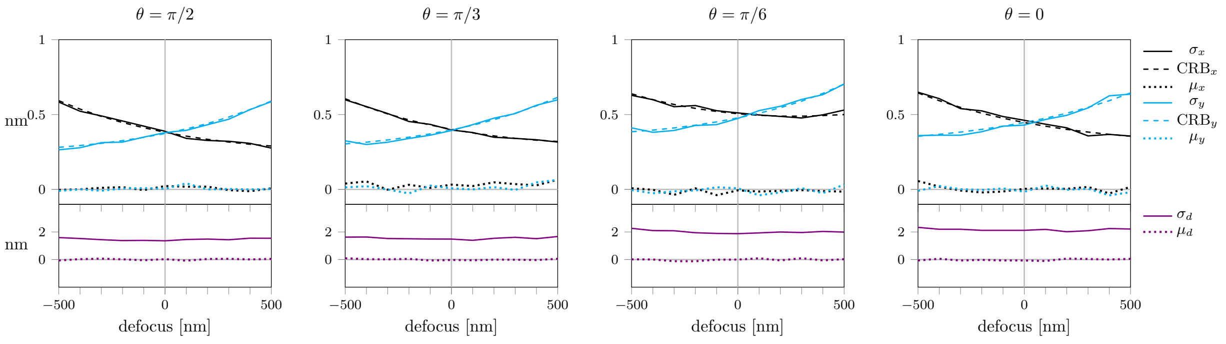

Figure S3

Localization errors in the presence of astigmatism. We fitted the position and defocus (x, y, d), while the dipole orientation (θ, ϕ) was assumed to be known exactly. All parameters were identical to Fig. 4, except for the azimuthal angle, which was set to $\phi = 0$. A list of all simulation parameters is given in Table S1.

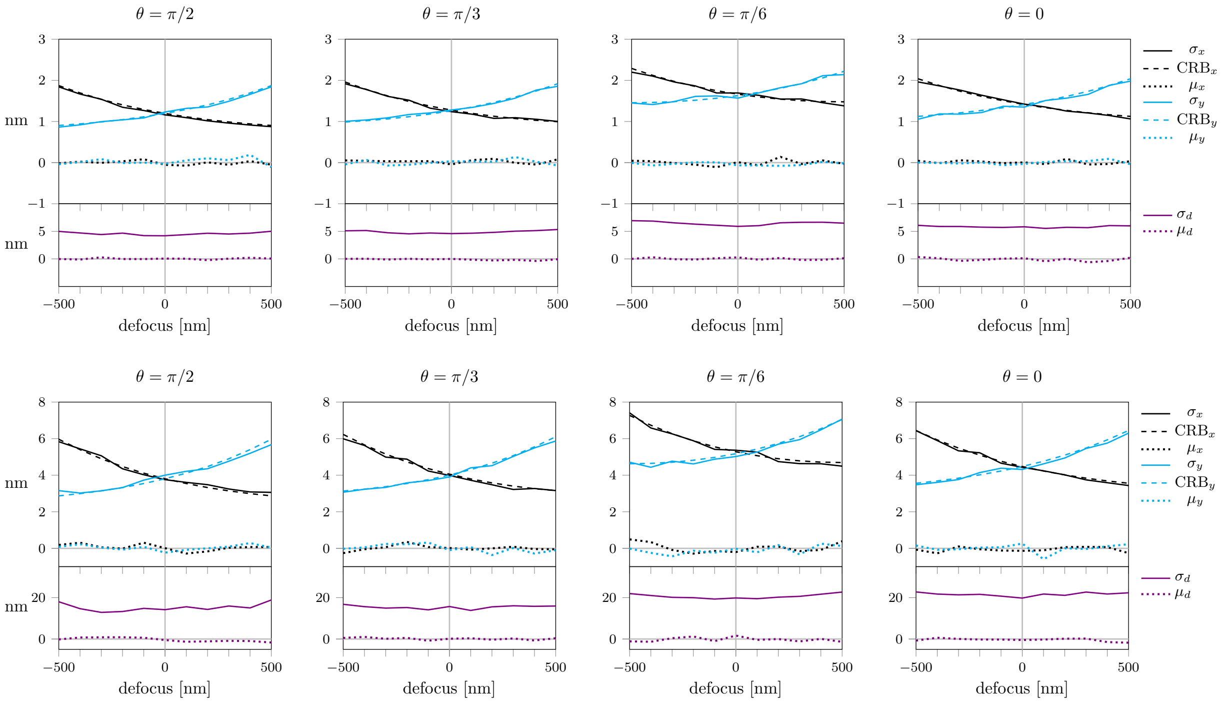

Figure S4

Localization errors in the presence of astigmatism. We fitted the position and defocus (x, y, d), while the dipole orientation (θ, ϕ) was assumed to be known exactly. All parameters were identical to Fig. 4, except for the photon number, which was reduced to $5 \cdot 10^4$ (first row) and further to $5 \cdot 10^3$ (second row). A list of all simulation parameters is given in Table S1.

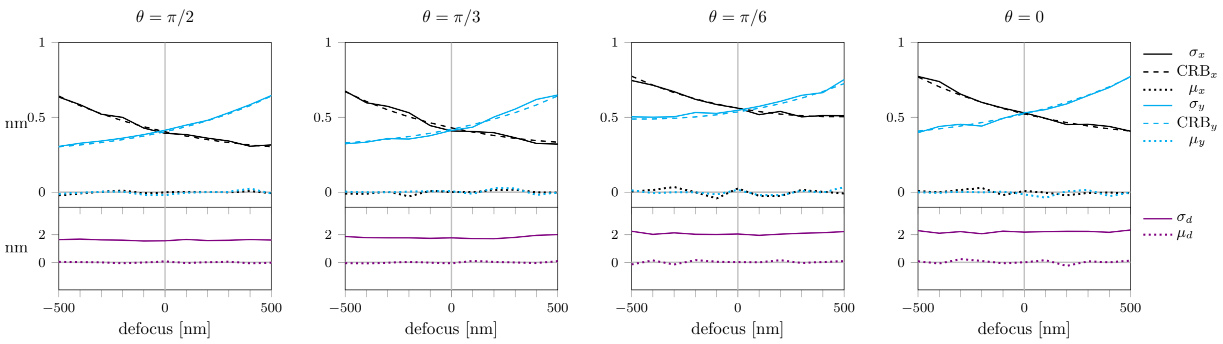

Figure S5

Influence of pixel size. All simulation parameters and the fitting procedure were identical to Fig. 4, except for the simulated pixel size, which was set to 216 nm. A list of all simulation parameters is given in Table S1.

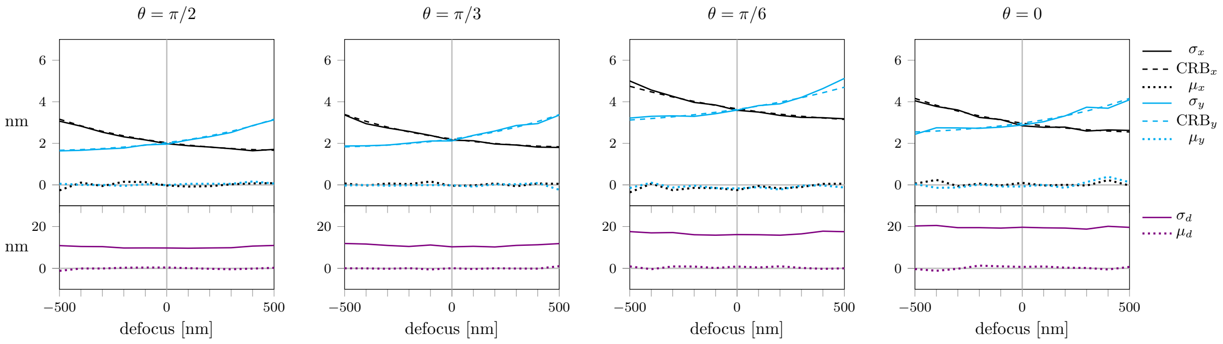

Figure S6

Influence of background noise. All simulation parameters and the fitting procedure were identical to Fig. 4, except for assuming background noise with $b = 300$. A list of all simulation parameters is given in Table S1.

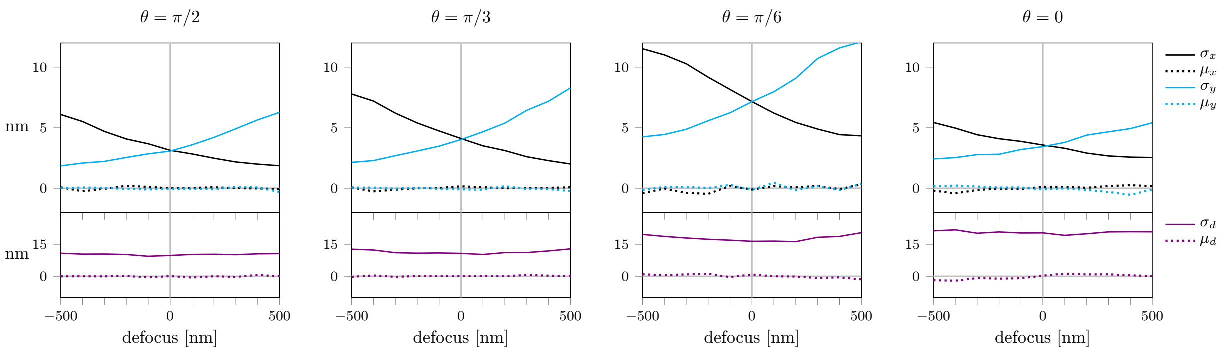

Figure S7

Influence of uncertainties in dipole orientation. Simulations and fitting procedure analogous to Fig. 4, except for a normal distribution of the error in orientation estimation with standard deviation of 2° for both θ and ϕ. Background noise was set to $b = 300$. A list of all simulation parameters is given in Table S1.

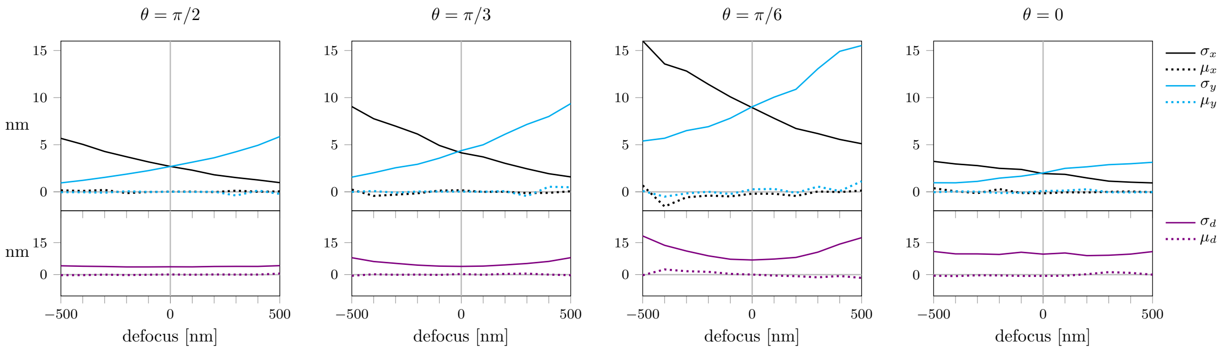

Figure S8

Influence of uncertainties in dipole orientation. Simulations and fitting procedure analogous to Fig. 4, except for a normal distribution of the error in orientation estimation with standard deviation of 4° in θ and 2° in ϕ. Background noise was set to $b = 100$. A list of all simulation parameters is given in Table S1.

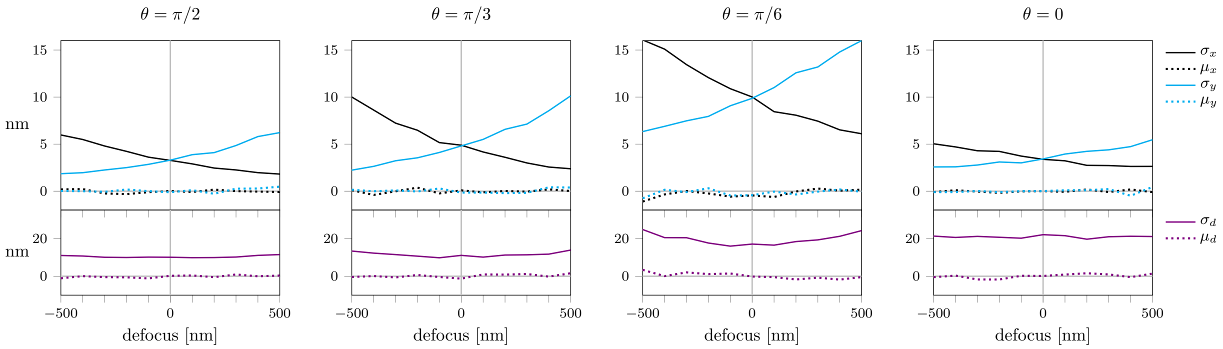

Figure S9
Influence of uncertainties in dipole orientation. Simulations and fitting procedure analogous to Fig. 4, except for a normal distribution of the error in orientation estimation with standard deviation of 4° in θ and 2° in ϕ. Background noise was set to $b = 300$. A list of all simulation parameters is given in Table S1.

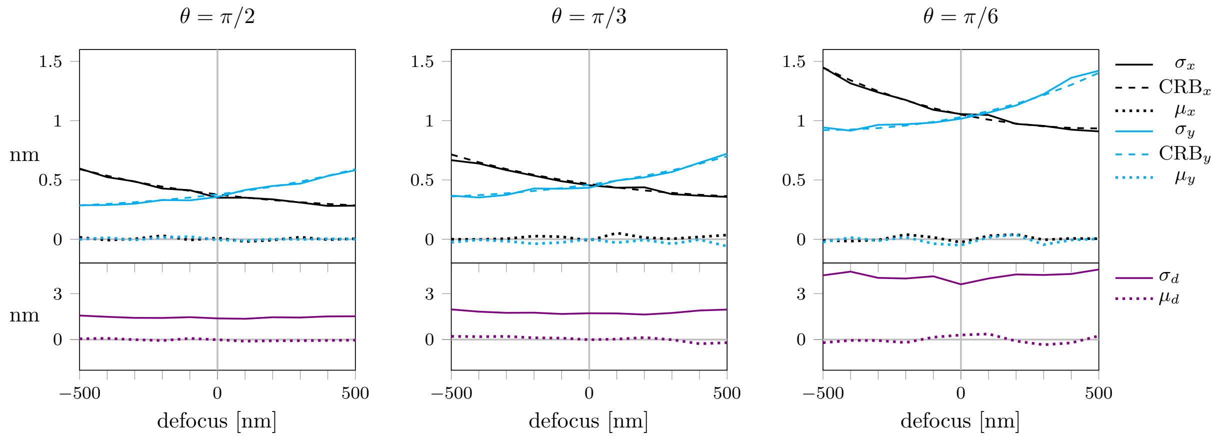

Figure S10
Influence of reduced excitation probability for tilted dipoles. Photon yield was adjusted depending on the dipole orientation with a maximum photon number of $5 \cdot 10^5$. The effective number of photons N_{eff} is indicated in each panel. The remaining parameters are identical to Fig. 4, of note background noise was set to $b = 0$. A list of all simulation parameters is given in Table S1.
