# Supplementary material for: Robust and bias-free localization of individual fixed dipole emitters achieving the Cramér Rao bound for applications in cryo-single molecule localization microscopy
Source: PLoS One. 2022 Feb 4;17(2):e0263500. doi: 10.1371/journal.pone.0263500 (PMC8815875; doi:10.1371/journal.pone.0263500)
Supplement: Attachment — Submitted filename: Response to Reviewers.pdf [file pone.0263500.s003.pdf]

Rebuttal letter

We thank the reviewer for the thoughtful inputs to our manuscript. In the revised version we addressed all raised points. Below, you find a point-by-point reply, including references to the changes in the manuscript. Note that line numbers refer to the manuscript with tracked changes. In addition, we corrected a few further typos in the manuscript.

Reviewer 1

Overall the paper is well written and the figures are well designed. It is a purely computational work, but with several interesting aspects. The manuscript deserves publication in PlosOne, but I ask the authors to address the following points:

Question: *As the approach of the work treats a particular problem of SMLM experiments at cryogenic temperatures, this should be mentioned in the abstract, probably also in the title.*

Answer: We thank the reviewer for this suggestion. We amended the title and abstract accordingly.

Question: *Fig. 1: Typo in caption (f)*

Answer: We corrected the wrong panel reference in Fig. 1.

Question: *Can the authors please justify why they have chosen half a million photons per emission, e.g. by referring to experimental evidence.*

Answer: Indeed, values above 10^6 for the number of obtained photons per fluorophore were reported previously for experiments performed under cryogenic conditions, due to decelerated photophysics. We included a short statement in lines 204–206 of the manuscript and added two references (Li, 2015 and Weisenburger, 2013).

Question: *How realistic is the chosen background noise (b = 300 photons standard deviation)?*

Answer: For data recorded at the cryo-setup in our own laboratory, we typically observe a background with a standard deviation of 10-12 photons per image. In practice, however, researchers may combine images until photobleaching of the fluorophore. In this case, background noise would increase with the square root of the added images. For example, to obtain a noise level of $b = 300$ one would need to add 900 frames; hence, the choice of $b = 300$ represents a rather high estimate for the noise in the data. We included a short statement in lines 206–209 of the manuscript.

Question: *Please also simulate photon numbers <10,000 photons, which would be realistic in classical SMLM experiments at RT. Figure S4 could be extended by one or two further panels.*

Answer: We included an additional panel row in Figure S4, including four new panels for the different dipole orientations simulated with $N = 5000$ photons. We refer to the new panel in line 252 of the manuscript.

Question: *Based on the proposed method, could any other 3D approaches be advantageously exploited, such as multiplane/biplane imaging?*

Answer: We thank the reviewer for this question. Indeed, also other 3D approaches will be helpful for determining the amount of defocus and, hence, also the correct lateral position of the fluorophores. We included a corresponding statement in lines 315–321 of the manuscript and added references (Deschout 2014, Backlund 2012, Hulleman 2021).

Question: *Although dipole moments of fluorophores employed in aqueous solution are considered to be freely rotating, are there any situations in which the suggested approach could be of use?*

Answer: Primarily, our approach is intended for applications in cryo-SMLM, where the mobility of molecules is prohibited and, hence, the orientation of fluorophore dipoles is fixed. At room temperature, fluorophore dipoles are typically freely rotating. However, for fluorophores with two attachment sites the mobility could be restricted. Of note, our approach only yields optimal results if the orientation of the fluorophore is fixed and does not allow for wobbling of the dipole orientation. We included a paragraph on the rotation of fluorophores in the Discussion Section in lines 332–337 of the manuscript.
